# Analysis of tau post-translational modifications in rTg4510 mice, a model of tau pathology

**DOI:** 10.1186/s13024-015-0011-1

**Published:** 2015-03-26

**Authors:** Lixin Song, Sherry X Lu, Xuesong Ouyang, Jerry Melchor, Julie Lee, Giuseppe Terracina, Xiaohai Wang, Lynn Hyde, J Fred Hess, Eric M Parker, Lili Zhang

**Affiliations:** Department of Neuroscience, Merck Research Laboratories, Kenilworth, NJ USA; Molecular Biomarkers, Merck Research Laboratories, Kenilworth, NJ USA; In Vivo Pharmacology, Merck Research Laboratories, West Point, PA USA; Current address: BioDuro, a PPD Company, Beijing, China; Current address: Medical Oncology Department, Rutgers Cancer Institute of New Jersey, New Brunswick, NJ USA; Current address: Human Oncology and Pathogenesis Program, Memorial Sloan Kettering Cancer Center, New York, NY USA; Current address: Department of Neuroscience, Novartis Institute for Biomedical Research, Cambridge, MA USA

**Keywords:** Alzheimer’s disease, Neurodegeneration, Tau, Tau aggregation, Tau phosphorylation, Tau acetylation, rTg4510, CSF tau

## Abstract

**Background:**

Microtubule associated protein tau is the major component of the neurofibrillary tangles (NFTs) found in the brains of patients with Alzheimer’s disease and several other neurodegenerative diseases. Tau mutations are associated with frontotemperal dementia with parkinsonism on chromosome 17 (FTDP-17). rTg4510 mice overexpress human tau carrying the P301L FTDP-17 mutation and develop robust NFT-like pathology at 4–5 months of age. The current study is aimed at characterizing the rTg4510 mice to better understand the genesis of tau pathology and to better enable the use of this model in drug discovery efforts targeting tau pathology.

**Results:**

Using a panel of immunoassays, we analyzed the age-dependent formation of pathological tau in rTg4510 mice and our data revealed a steady age-dependent accumulation of pathological tau in the insoluble fraction of brain homogenates. The pathological tau was associated with multiple post-translational modifications including aggregation, phosphorylation at a wide variety of sites, acetylation, ubiquitination and nitration. The change of most tau species reached statistical significance at the age of 16 weeks. There was a strong correlation between the different post-translationally modified tau species in this heterogeneous pool of pathological tau. Total tau in the cerebrospinal fluid (CSF) displayed a multiphasic temporal profile distinct from the steady accumulation of pathological tau in the brain. Female rTg4510 mice displayed significantly more aggressive accumulation of pathological tau in the brain and elevation of total tau in CSF than their male littermates.

**Conclusion:**

The immunoassays described here were used to generate the most comprehensive description of the changes in various tau species across the lifespan of the rTg4510 mouse model. The data indicate that development of tauopathy in rTg4510 mice involves the accumulation of a pool of pathological tau that carries multiple post-translational modifications, a process that can be detected well before the histological detection of NFTs. Therapeutic treatment targeting tau should therefore aim to reduce all tau species associated with the pathological tau pool rather than reduce specific post-translational modifications. There is still much to learn about CSF tau in physiological and pathological processes in order to use it as a translational biomarker in drug discovery.

**Electronic supplementary material:**

The online version of this article (doi:10.1186/s13024-015-0011-1) contains supplementary material, which is available to authorized users.

## Background

Microtubule associated protein tau is expressed primarily in neurons and plays an important role in axonal transport [1]. Abnormal accumulation of tau in the brain of patients with Alzheimer’s disease (AD) leads to the formation of neurofibrillary tangles (NFTs), which together with β-amyloid plaques are the two pathological hallmarks of the disease [2,3]. While plaques can be found in post-mortem brains from people without significant clinical AD symptoms, the presence of NFTs correlates well with neuronal cell death and the loss of cognitive functions. In addition, NFTs are also found in the brains of patients with several other neurodegenerative diseases that lack amyloid pathology [2]. Understanding the development of tau pathology, also known as tauopathy, should provide important insights into the etiology of AD and other neurodegenerative diseases, and into the development of therapeutic strategies targeting this pathway.

Human tau exists as six alternatively spliced isoforms, with its C-terminal half containing either 3 or 4 repeats of the microtubule binding domain (designated as either 3R or 4R tau). In adult brain under normal physiological conditions, the 3R and 4R tau are present in about a 1:1 ratio. Pathological tau in AD brain is hyperphosphorylated and forms insoluble aggregates that eventually develop into NFTs [2]. This observation has led to the hypothesis that abnormal phosphorylation plays a major role in the disease process. In addition to phosphorylation, tau undergoes multiple post-translational modifications such as acetylation, nitration, ubiquitination, etc. [4,5]. Moreover, tau is also present in cerebral spinal fluid (CSF) and tau in CSF is elevated in AD patients long before the clinical symptoms of the disease are manifest [6]. These findings suggest that the development of tauopathy is dependent on far more than just hyperphosphorylation. The biological and pathological processes involved in tau post-translational modifications and CSF tau production are largely unknown.

The identification of tau mutations associated with frontotemporal dementia with parkinsonism on chromosome 17 (FTDP-17) [7,8] establishes the pathogenic role of tau in mediating neurodegeneration and cognitive decline. FTDP-17 mutations affect the tau microtubule binding or alternative splicing in favor of the 4R form of tau (reviewed by [1]). Many transgenic animal models have been generated to explore the mechanism of tauopathy development. Transgenic models expressing human tau with missense FTDP-17 mutations display more robust NFT formation compared to those that express wild-type human tau [9]. One of the models, the rTg4510 mouse, overexpresses a human tau transgene carrying a P301L mutation, with transgene expression driven by the Ca^2+^-calmodulin kinase II promoter [10]. NFT staining can be detected in rTg4510 mice as early as 5–6 months of age and, like NFT pathology in AD, is restricted to the forebrain structures such as hippocampus and cortex. This model also develops neuronal cell loss, brain atrophy and cognitive decline reminiscent of that seen in AD patients [10,11], making it an attractive model to investigate disease mechanisms and test potential therapeutic agents.

Here we report the development of a comprehensive panel of sandwich immunoassays to study the tauopathy in rTg4510 mice. The sensitivity and quantitative nature of these assays enabled us to investigate the correlations between different tau species over the lifespan of the rTg4510 mice, thereby providing important information on the properties of pathological tau and the time course of its development in this model. These findings will guide the effective use of rTg4510 mice in studies of the basic biology of AD and other tauopathies, and in the assessment of potential treatments for these conditions.

## Results

**Figure 1:**
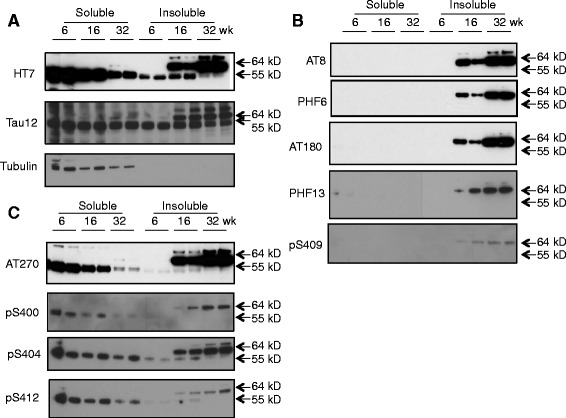
**Western blot analysis of various tau species in brain lysates from Tg4510 mice of different ages.** Soluble and insoluble fractions of brain homogenates were prepared as described in Methods from rTg4510 mice that were 6, 16 or 32 weeks of age. Ten μg of protein from each fraction was analyzed by probing Western blots with the specified antibodies. The position of the 55 kD and 64 kD species of tau are indicated. α-Tubulin was used as a loading control. **A**. Total tau detected by two different antibodies (HT7 and Tau12). **B**. P-tau species enriched in 64 kD tau in the insoluble fraction. **C**. P-tau species present in both the 55 kD species in the soluble fraction and in the 64 kD species in the insoluble fraction.

To study changes in tau during disease progression in rTg4510 mice, we separated the brain homogenates into soluble and insoluble fractions by a simple two step centrifugation process based on a previously reported study [12]. Western blots probed with HT7 and tau-12 antibodies, which recognize epitopes independent of phosphorylation and detect total tau, revealed an age-dependent decrease of total tau in the soluble fraction and an age-dependent increase of total tau in the insoluble fraction. Tau in the insoluble fraction also underwent a transition from a 55 kD species to a higher molecular weight 64 kD species as the animals aged (Figure 1A). There were two distinct profiles of phosphorylated tau (p-tau) changes during disease progression in the rTg4510 mice. One type of p-tau, as detected by antibodies AT8, PHF6, AT180, PHF13 and pS409, was enriched in the 64 kD form in the insoluble fraction and increased in the insoluble fraction as the animals aged (Figure 1B). The second type of p-tau, as detected by antibodies AT270, pS400, pS404 and pS412, displayed a profile similar to that of total tau, i.e., a decrease in the soluble fraction, an increase in the insoluble fraction and a transition from a 55 kD to a 64 kD species in the insoluble fraction as the animals aged (Figure 1C). These data are consistent with observations from other groups and demonstrate an age-dependent development and accumulation of a 64 kD species of pathological tau that is enriched in the insoluble fraction of brain extracts and correlates with the development of tauopathy, neuronal cell loss, brain atrophy and cognitive impairment [10].

**Figure 2:**
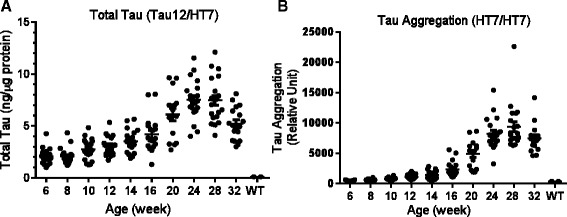
**Age-dependent change of total tau and tau aggregates in the brain insoluble fraction of rTg4510 mice.** Brain insoluble fractions from animals 6–32 weeks of age (n = 18-20 per group) were analyzed using AlphaLISA based immunoassays. **A**. Total tau measured in an immunoassay using biotin-Tau12 for capture and HT7-conjugated acceptor beads for detection. Twenty ng of protein from the insoluble fractions were used per assay. Recombinant tau (Millipore) was used to generate a standard curve for quantification. **B**. Tau aggregates measured in an immunoassay using HT7 as both capture and detection antibody. Two hundred ng of protein from the insoluble fractions were used in each assay. The Y-axis is the relative fluorescence readout from Envision (PerkinElmer). Compared to 8-week old mice, total tau and tau aggregate changes reached statistical significance after 14-, and 16-weeks of age, respectively (p <0.05, one-way ANOVA).

**Figure 3:**
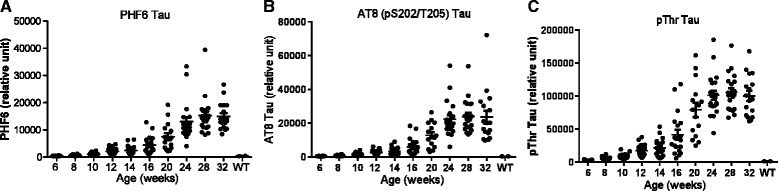
**Age-dependent change of p-tau in the brain insoluble fraction of rTg4510 mice.** Brain insoluble fractions from animals 6–32 weeks of age (n = 18-20 per group) were analyzed using AlphaLISA based immunoassays. PHF6 tau **(A)**, AT8 tau **(B)**, and pThr global tau phosphorylation **(C)** were measured using biotin-HT7 for capture and specific p-tau antibodies for detection. Two hundred ng of protein from the insoluble fractions were used in each assay. The Y-axis is the relative fluorescence readout from Envision (PerkinElmer). Compared to 8-week old mice, PHF6, AT8 and pThr tau changes reached statistical significance after 16-, 20-, and 16-week of age, respectively (p <0.05, one-way ANOVA).

Next we focused on the change of tau in the insoluble fraction in which the pathological 64 kD tau species was enriched. A panel of sensitive sandwich immunoassays was developed using AlphaLISA technology (PerkinElmer) to analyze different tau species during the progression of tauopathy in rTg4510 mice (Table 1). An age-dependent accumulation of both total tau and aggregated tau was observed in the insoluble fraction (Figure 2A and 2B). It is noteworthy that large inter-animal variations in both total and aggregated tau were observed within each age group, particularly at older ages where tauopathy was progressing rapidly and significant amounts of tau were accumulating in the insoluble fraction. The various species of p-tau studied also showed an age-dependent increase in the insoluble fraction, including p-tau species detected by PHF6 (conformation around pT231; Figure 3A), AT8 (pS202/T205; Figure 3B), pS262, pS400, AT180 (pT231), PHF13 (pS396), AT100 (pS212/pT214) and pS409 (Additional file 1: Figure S1). We also developed an immunoassay for global tau phosphorylation using a pan anti-phosphothreonine antibody and showed that, similar to other p-tau species, pThr global tau phosphorylation was also elevated in the insoluble fraction (Figure 3C). For most of the tau species in the soluble fraction, the age-dependent increase reached statistical significance at 16 weeks as compared to 8 weeks of age (one-way ANOVA, p < 0.05, Figures 2 and 3, Additional file 1: Figure S1).

**Table 1:** **List of tau species detected and antibody reagents used in the AlphaLISA immunoassays developed for this study**

**Tau Species**	**Biotin-donor**	**Acceptor**
Total tau	Tau12	HT7
Tau aggregates	HT7	HT7
p231	HT7	PHF6
pT202/205	HT7	AT8
Global p-tau	HT7	anti-pThr
pT231	HT7	AT180
pT181	HT7	AT270
pS212/T214	HT7	AT100
pS262	HT7	pS262
pT396	HT7	PHF13
pS400	HT7	pS400
pS404	HT7	pS404
pS409	HT7	pS409
pS412	HT7	pS412
Ac-Lys280	HT7	Anti-Ac-Lys280
Ac-tau	HT7	anti-acetyl-Lys
pY-tau	HT7	anti-p-tyrosine
ub-tau	HT7	anti-ubiquitin
nY29 tau	HT7	anti-nY29
CSF total tau	HT7	BT2

**Figure 4:**
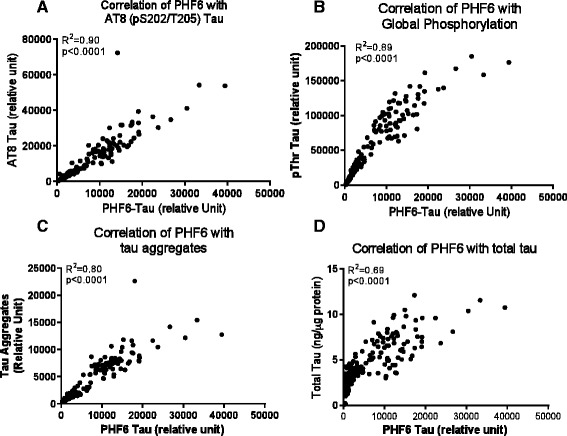
**Correlation of various tau species in the insoluble fraction of rTg4510 mouse brain.** The data in Figures 2 and 3 were analyzed for correlation with PHF6 tau using Prism 5.0 software. Shown are correlations of PHF6 tau with: **A**. AT8 tau; **B**. pThr tau global phosphorylation; **C**. tau aggregates; and **D**. total tau.

A further analysis revealed that despite the large variations between animals, the levels of all p-tau species showed strong correlation with the levels of the p-tau species recognized by PHF6, including AT8 (R^2^ = 0.9, p < 0.0001, Figure 4A), pThr global phosphorylation (R^2^ = 0.89, p < 0.0001, Figure 4B), pS262 (R^2^ = 0.82, p < 0.0001), pS400 (R^2^ = 0.88, p < 0.0001), AT180 (R^2^ = 0.93, p < 0.0001), PHF13 (R^2^ = 0.9, p < 0.0001), AT100 (R^2^ = 0.94, p < 0.0001) and pS409 ((R^2^ = 0.8, p < 0.0001, Additional file 2: Figure S2). In addition, a significant correlation was observed between PHF6 tau and tau aggregates (R^2^ = 0.80, p < 0.0001, Figure 4C) and between PHF6 tau and total tau (R^2^ = 0.69, p < 0.0001, Figure 4D) in the insoluble fraction.

**Figure 5:**
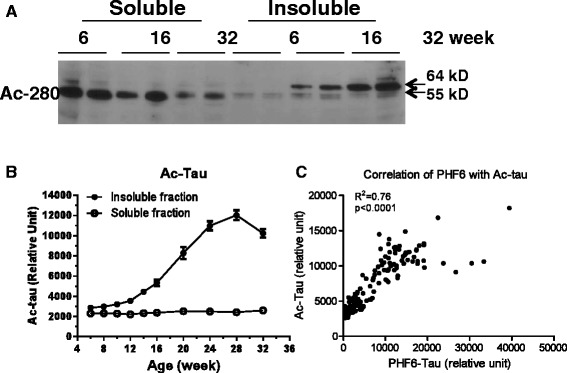
**Acetylated tau in rTg4510 brain. A**. Western blot of acetylated tau probed by ac-280, a polyclonal antibody specific for tau acetylated at Lys280. **B**. Age-dependent change of acetylated tau in the soluble and insoluble fractions of rTg4510 mouse brain. Acetylated tau was measured using 1 μg of protein from the soluble or insoluble fractions of rTg4510 mouse brain with biotin HT7 for capture and a pan acetyl-lysine antibody for detection. The error bars represent the standard error for each group. The change of ac-tau reached statistical significance at 14-weeks of age as compared to 8-weeks (p < 0.05, one-way ANOVA). **C**. Correlation of the levels of acetylated tau and PHF6 tau in the insoluble fraction of rTg4510 mouse brain.

Tau acetylation has recently been reported to be increased in the brains of tau transgenic mice and in the brains of AD patients [13,14]. Western blots probed with Ac-280 showed this tau species acetylated at Lys280 migrated with the 55 kD tau species in the soluble fraction from young rTg4510 mice but shifted to the 64 kD species in the insoluble fraction as tauopathy progressed (Figure 5A). An immunoassay to assess global tau acetylation was developed that utilized a pan anti-acetylated lysine antibody in combination with antibody HT7. This immunoassay confirmed that the global tau acetylation was much enriched in the insoluble fraction as compared to the soluble fraction. The acetylated tau was elevated as the rTg4510 animals aged and the change reached statistical significance at the age of 14-weeks as compared to 8-weeks (p < 0.05, one-way ANOVA, Figure 5B). Interestingly, acetylated tau also showed a strong correlation with PHF6 tau in the insoluble fraction (R^2^ = 0.76, p < 0.0001, Figure 5C), suggesting that both modifications are present in the same pool of tau that contribute to tauopathy and neurodegeneration in this model.

**Figure 6:**
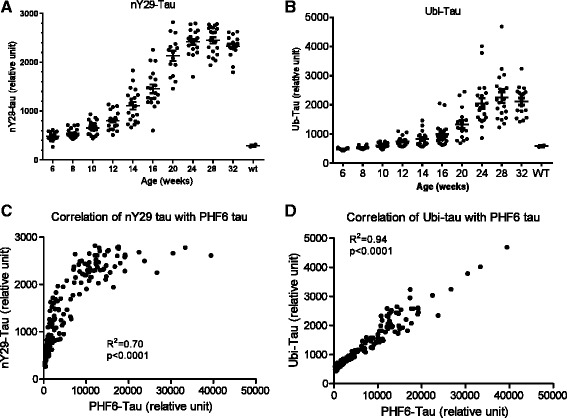
**Age-dependent change of ubiquitination and nitration of tau in the brain insoluble fraction of rTg4510 mice.** Post-translational modifications were measured using AlphaLISA immunoassays with 1 μg of protein from the brain insoluble fraction of rTg4510 mice. **A**. Age-dependent change of tau ubiquitination. Ub-tau was detected using biotin HT-7 for capture and an anti-ubiquitin antibody for detection. **B**. Age-dependent change of Tyr29 nitration of tau. nY29 tau was detected using biotin HT-7 for capture and an anti-nY29 antibody for detection. **C**. and **D**. Correlation of Ub-tau **(C)** and nY29 tau **(D)** with PHF6 tau. Correlation analysis was done using Prism 5.0 software.

Next we examined ubiquitination and tyrosine nitration of tau in rTg4510 mice, both of which have also been shown to be associated with NFT pathology [15-17]. As seen with multiple p-tau species and acetylated tau, both ubiquitinated tau (ub-tau) and tau nitrated at tyrosine 29 (nY29 tau) were enriched in the insoluble fraction and showed age-dependent increases in rTg4510 mice (Figure 6A and B). We did not detect either ub-tau or nY29 tau in the soluble fraction using the assays described here (data not shown). Compared to 8-week old mice, the changes of ub-tau and nY29 tau reached statistical significance at the age of 12 and 16-weeks, respectively (p < 0.05, one-way ANOVA, Figure 6A and B). Again, the change of both tau species correlated strongly with the change of PHF6 tau during the development of tauopathy (R^2^ = 0.70, p < 0.0001 for nY29 and R^2^ = 0.94, p < 0.0001 for ub-tau, Figure 6C and D).

**Figure 7:**
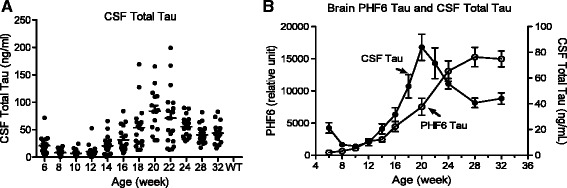
**Age-dependent change of CSF total tau in rTg4510 mice.** CSF total tau was measured using an AlphaLISA assay with biotin HT7 antibody for capture and antibody BT2 conjugated acceptor beads for detection. One microliter of CSF was used per assay and recombinant tau (Milipore) was used to generate a standard curve for quantification. **A**. Age-dependent change in CSF total tau in Tg4510 mice. **B**. Re-plotting of age-dependent changes in mean brain PHF6 tau levels from Figure 3A (open circle) and mean CSF tau levels from Figure 7A (solid circle). Error bars represent the standard error of the mean.

Similar to the various tau species comprising pathological tau in the brain, there was a large inter-animal variation in CSF tau as rTg4510 mice age (Figure 7A). However, unlike the steady accumulation of total tau and various p-tau species in brain as rTg4510 mice age (e.g. as illustrated by PHF6 tau levels in Figure 7B), levels of total tau in CSF of rTg4510 mice changed in a multi-phasic pattern during aging. CSF total tau consistently decreased from 6 to 8–12 weeks of age, although the decrease was not statistically significant. Subsequently, CSF total tau progressively increased, with the increase reaching statistical significance at 16 weeks of age and beyond (one-way ANOVA, p < 0.05). After reaching a peak at 20 weeks of age, CSF total tau then showed a steady decline that was statistically significantly from 24–32 weeks of age (p < 0.05, one-way ANOVA) (Figure 7B).

**Figure 8:**
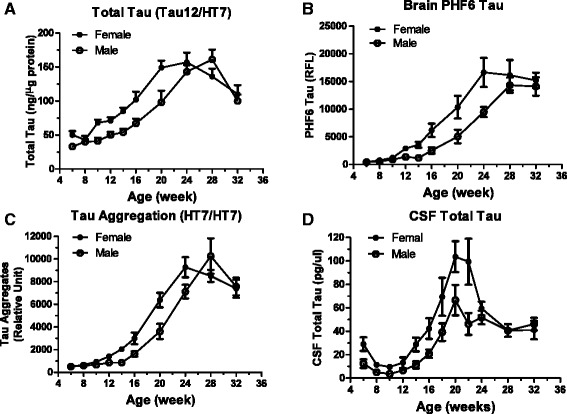
**Gender difference in rTg4510 mice.** Data from previous figures were plotted separately for male (open circle) and female (solid circle) mice. Error bars represent the standard error of the mean. Factorial ANOVAs were used to analyze the brain and CSF tau data for male and female mice at various ages with sex and age as the between subjects factors. **A**. Total Tau. Total tau levels were greater in the brains of female mice compared to males [main effect of sex, F(1,166) = 19.43, p < 0.0001], total tau levels varied as the mice aged and the age-related variation was different for females compared to males [Sex x Age interaction, F(9,166) = 2.43, p < 0.02] with the males catching up to the females by 24 weeks of age. **B**. PHF6 Tau. Female mice had higher levels of PHF6 tau compared to males [main effect of Sex, F(1,172) = 18.78, p < 0.0001] and PHF6 tau levels increased as the mice aged. **C**. Tau Aggregation. Female mice showed greater tau aggregation than males [main effect of Sex, F(1,170) = 6.59, p < 0.02], tau aggregation increased and peaked as the mice aged and the age-related variation was different for females compared to males [Sex x Age interaction, F(9,170) = 2.56, p < 0.009] with the tau aggregation peaking earlier in females than males (24 weeks vs. 28 weeks). **D**. CSF Total Tau. Female mice had higher CSF total tau levels compared to males [main effect of Sex, F(1,197) = 21.24, p < 0.0001], CSF total tau levels varied as the mice aged and the difference between females and males varied at different ages [Sex x Age interaction, F(11,197) = 2.31, p < 0.02] with similar levels being observed in females and males at older ages.

When gender difference was assessed, female Tg4510 mice had an overall higher level of total tau (main effect of sex, F(1,166) = 19.43, p < 0.00010), tau aggregates (main effect of sex, F(1,170) = 6.59, p < 0.02) and PHF6 tau (main effect of sex, F(1,172) = 18.78, p < 0.0001) as the mice aged; however, rTg4510 mice of both genders reached similar peak values at 30–32 weeks of age (Figure 8A-C). Female Tg4150 mice also had higher CSF total tau levels during aging as compared to male mice [main effect of sex, F(1,197) = 21.24, p < 0.0001], but eventually similar levels of CSF total tau were observed in females and males at ages >24 weeks (Figure 8D).

## Discussion

rTg4510 mice express human tau with the P301L mutation and provide many advantages to study tauopathy, including a rapid onset of NFT formation and a brain distribution of NFTs mirroring that seen in AD patients. The development of tauopathy in this model has been well characterized in several studies [10-12,18]. We report here using an immunoassay platform to analyze different tau species in rTg4510 mouse brain. The higher throughput of this platform enabled us to study larger groups of animals to mitigate the large inter-animal variations in the time course and extent of tauopathy in this model. The sensitivity and the quantitative nature of these assays also allowed us to more precisely investigate the relationships between different features of tauopathy.

One of the characteristic features of NFT pathology in the brains of AD patients is the accumulation of hyperphosphorylated tau aggregates [2]. Many of the serine and threonine residues in tau have been reported to be phosphorylated in the brains of AD patients [4]. The hyperphosphorylation of tau is associated with a transition of tau from a predominantly soluble, lower molecular weight species to a predominantly insoluble, higher molecular weight species, a transition that is thought to be intimately associated with the development of tauopathy and neurodegeneration [2]. Consistent with previous reports [10,19], our data showed that as rTg4510 mice age, tau progressively transitions from a 55 kD species found predominantly in the soluble fraction of brain homogenates to a 64 kD species found predominantly in the insoluble fraction. Interestingly, some p-tau species are present in both the soluble and insoluble fractions of rTg4510 brain homogenates, while other p-tau species are primarily present in the insoluble fraction. However, both are present in the insoluble, 64 kD form of tau that is thought to be associated with the development of tauopathy (Figure 1). Thus, the 64 kD tau cannot be said to comprise only one or even a few p-tau species, but rather encompasses the global phosphorylation of tau at all epitopes studied. These data also suggest that the accumulation of 64 kD tau in the insoluble fraction can be used as a surrogate marker to monitor tauopathy development and that a treatment reducing pathological tau formation should lead to a correlative reduction of all tau species associated with tauopathy. Although NFT histology is typically first observed at 4–5 months of age, our biochemical analysis points to a steady accumulation of PHF6 and other pathological forms of tau starting from the young age of 6–8 weeks, with the changes reaching statistical significance by around 16–20 weeks. This finding points to the possibility for the effective use of the rTg4510 model to assess prophylactic treatments targeting tauopathy at ages as early as 6–8 weeks and for durations of treatment as short as 8–10 weeks.

In addition to phosphorylation, several additional post-translational modifications are also associated with pathological tau [4,5]. Like many species of p-tau, several other post-translationally modified forms of tau increased in the insoluble fraction of brain homogenates as rTg4510 mice aged, including tau aggregates, ac-tau, ub-tau and nY29 tau. Acetylated tau also transitioned from a predominantly soluble 55 kD species to an insoluble 64 kD species in rTg4510 brain homogenates as the animals aged (Figure 6A). These data provide the first comprehensive characterization of the behavior of these more recently described post-translationally modified forms of tau in the rTg4510 mouse model.

Our data demonstrated a close correlation between all the post-translationally modified species of tau examined in this study (Figures 4, 5 and 6, Additional file 2: Figure S2), suggesting these modifications are not separate and unrelated events but may collectively contribute to the disease process. Pathological tau thus represents a heterogeneous pool of mis-folded proteins encompassing tau phosphorylated at multiple epitopes as well as multiple additional post-translationally modified tau species. These post-translational modifications may occur more or less simultaneously as tauopathy develops and it is difficult to distinguish which one(s) (aggregation, phosphorylation, acetylation, etc.) may be the primary driver of the development of pathological tau. Further investigation in these areas is critical to understand the disease mechanism in the rTg4510 model and assess therapeutics targeting tauopathy.

Some of the correlations between different post-translationally modified forms of tau, although significant, are not linear. This is likely due to the technical limitation that we cannot assess assay linearity without a standard for each tau species. Therefore, the sample loadings in the analyses were determined by dilution linearity of the middle-aged samples and thus may not cover the wide range of tau levels in the different age groups. The lack of assay standards for each specific tau species also means we cannot determine the absolute concentration and stoichiometry of each species in this pool of pathological tau. However, this limitation does not impact the conclusion that all post-translational modifications of tau studied are all temporally associated with the development of tauopathy.

Elevation of CSF total tau is a characteristic feature of AD and is being explored as a diagnostic biomarker and as a biomarker to identify patients in clinical trials of potential AD therapeutics [6]. The elevation of CSF tau was first hypothesized to be a consequence of neuronal cell death, but this hypothesis is unlikely to explain the presence of tau in the CSF of healthy individuals of all ages. The emerging evidence that tau is released from normal cells and neurons [20-22], possibly as a mechanism of removing excessive cellular metabolites [23], provides an alternative hypothesis of the source of CSF tau. In rTg4510 mice, CSF tau changes in several phases as the animals age, a temporal pattern that is distinct from the steady increase of brain tauopathy (Figure 7B). Our findings are consistent with a previous report [24] and suggest that CSF tau levels in this model are likely determined by both normal tau metabolism (e.g. constitutive secretion by cells) and by processes associated with pathological tau formation. Further understanding the nature of age-dependent changes in CSF tau may lead to important insights into the mechanism of tauopathy progression and biomarker development for AD therapy.

Although there is a clear trend of pathological tau development in Tg4510 mice, the accumulation of pathological tau displays large variations in all parameters assessed. These variations are consistent with findings by others and are not due to differential transgene expression ([24], L. Song and L. Zhang unpublished data). It has been reported previously that female rTg4510 mice display more robust tau pathology and more severe deficits in spatial learning and memory tests [25]. Our data provide further evidence for a gender difference in that female rTg4510 mice showed significantly more aggressive age-dependent accumulation of pathological tau in all features we assessed (Figure 8A-D). The underlying mechanism for the gender difference is not clear but our observations provide important information for using this model in terms of gender selection or balance and group size to give sufficient power for target effect size.

## Conclusion

Our data demonstrate that tauopathy in rTg4510 mice develops via accumulation of a pool of pathological tau that carries multiple post-translational modifications, a process that can be detected well before the histological detection of NFTs and is more pronounced in female rTg4510 mice. Although hyperphosphorylation is the best known characteristic of tauopathy, there are several additional post-translationally modified forms of tau (e.g. acetylation, ubiquitination, nitration) that correlate equally well with formation of pathological tau and also temporally correlate with formation of hyperphosphorylated tau. Therefore, it is not clear which of the post-translational modification events is the proximal cause of pathological tau formation. Nevertheless, the data suggests that a treatment that reduces pathological tau formation should lead to a correlative reduction of all tau species associated with tauopathy. CSF tau in rTg4510 mice is likely derived from both normal metabolism and the formation of pathological tau and exhibits a complex temporal pattern that is distinct from the progressive increase in pathological tau formation in the brain. Further understanding of the biological and pathological processes of CSF tau production remains an important aspect for future studies given its importance as a translational biomarker of efficacy and patient selection for preclinical and clinical testing of potential treatments targeting tauopathies. In summary, this study provides several important insights into the development of tauopathy in rTg4510 mice and into the use of this model to assess potential therapeutic treatments targeting tau pathology.

## Methods

### Reagents

Antibodies used for Western blots and AlphaLISA assays are listed in Table 2. Ac-280 is a custom rabbit polyclonal antibody developed against an acetylated tau peptide as described [14]. The serum was pre-absorbed with an un-modified tau peptide of the same amino acid sequence before being affinity purified using the acetylated peptide (GenScript). The specificity of ac-280 was confirmed by Western blots showing that this antibody selectively picked up hyper-acetylated tau when tau was co-transfected with histone acetyltransferase P300 in HEK293 cells (Additional file 3: Figure S3).

**Table 2:** **Antibody reagents used in this study**

**Antibody**	**Epitope**	**Source**
HT7	aa159-163	Thermo Scientific
BT2	aa194-198	Thermo Scientific
Tau12	N-terminus	Covance
AT270	pT181	Thermo Scientific
AT180	pT231	Thermo Scientific
AT100	pS212/T214	Thermo Scientific
PHF6	PHF around pT231	Covance
AT8	pS202/T205	Thermo Scientific
PHF13	PHF pT396	Covance
PS400	phospho-S400	Thermo Scientific
PS404	phospho-S404	Invitrogen
PS409	phospho-S409	Invitrogen
PS412	phospho-S412	AnaSpec
p-Thr	pan phosphor-threonine	Sigma
Ac-Lys	pan acetyl-lysine	Cell Signaling
Ac-280	aa264-287 with acetyl-Lys280	Prepared as described in [13]
Ubiqutin	pan ubiquitin	Millipore
nY29	nitrated tyrosine 29	Abcam

### Animals

The rTg4510 mouse line overexpressing the human tau 0N4R isoform carrying the P301L mutation [10] was licensed from the Mayo Clinic and bred at Taconic in Germantown, NY. The mice were bred by crossing the hTau responder line (FVB/N strain) and the tTA activator line (129S6 strain) as described [10]. All animal procedures are in compliance with the NIH Guide for the Care and Use of Laboratory Animals and were approved by the Institutional Animal Care and Use Committee of Merck Research Laboratories, an AAALAC accredited institution.

Animals of different ages (gender balanced, n = 18–20 per group with the exception of 12 at 6 weeks of age and 15 at 20 weeks of age) were euthanized with CO_2_. Brain and CSF were collected from each animal. For CSF collection, mice were euthanized and placed on a table with the head tilted down 70–80 degrees. The skin was removed to expose muscles located between the ears, the caudal part of head and the rostral neck to locate the cisterna magna. A 27G butterfly needle connected to a 1 ml syringe was used to punch through the muscle to the cisterna magna and about 10 μL CSF was collected by slightly pulling the syringe.

### Preparation of brain fractions

The soluble and insoluble fractions of rTg4510 mouse brain were prepared based on a previously published method with some modifications [12]. Briefly, half brains from rTg4510 or wild-type mice were homogenized using metal beads with TissueLyzer (Qiagen) in 900 μL preparation buffer containing 50 mM Tris, pH 8.0, 250 mM NaCl, 5 mM KCl, 2 mM EDTA, 2 mM EGTA, PhosphoSafe extraction buffer (EMD/Novagen) plus Complete EDTA-free Protease Inhibitor Tablet (Roche), PhoStop Tablet (Roche), 2 μM Trichostatin A (Sigma-Aldrich), 5 mM Nicotinamide (Sigma-Aldrich), and 1 μM PUGNAc (Sigma-Aldrich). The homogenates were centrifuged at 14,000 g for 15 min to remove the tissue debris. The supernatants were then centrifuged at 100,000 g for 30 min. The pellets were re-suspended in the preparation buffer, and the supernatants and the pellets from the second spin were defined as the soluble and insoluble fraction, respectively. Protein concentrations were determined using the BCA assay kit (Pierce).

### AlphaLISA-based sandwich immunoassays

A comprehensive panel of sandwich immunoassays listed in Table 1 was developed to measure different forms of tau. In general, analytes were captured by a monoclonal antibody that was biotinylated and bound to streptavidin coated donor beads (PerkinElmer). Detection was accomplished either by a monoclonal antibody conjugated to the acceptor beads directly or by a polyclonal antibody in combination with anti-rabbit IgG-conjugated acceptor beads (PerkinElmer).

Assay reactions (25 μl) were carried out in OptiPlate-384 microplates (PerkinElmer) that contained 5 μL of analyte at the specified protein concentration, 10 μL of biotinylated capture antibody (final concentration 2 nM) and 10 μL of detection antibody-conjugated acceptor beads (final concentration 20 μg/mL). After overnight incubation at 4°C, 25 μL of streptavidin donor beads were added under subdued light conditions (final concentration 40 μg/mL) and the reactions were incubated at room temperature for 60 min with gentle shaking. The fluorescent signal was detected on an Envision Plate Reader at 615 nm (PerkinElmer).

### Total tau and tau aggregates

Total tau assays were defined as detection of tau using antibodies recognizing epitopes not affected by phosphorylation. Here we used biotin-Tau 12 with HT7 conjugated acceptor beads for analyzing brain total tau, and biotin-HT7 with BT2 conjugated acceptor beads for analyzing CSF total tau. Recombinant tau (Millipore) was used to generate a standard curve for quantification. In addition, biotin-HT7 was also used in combination with HT7 conjugated acceptor beads for analyzing aggregated forms of tau. This assay detects any tau aggregate that is a dimer or larger, and thus does not distinguish between soluble oligomeric and insoluble, higher order aggregates of tau. Formic acid treatment, which denatures tau aggregates, abolished the signal in this HT7-HT7 assay (data not shown). The total tau assay detects both 55 and 64 kD tau species while the aggregated tau assay preferentially detects the 64 kD species that is enriched in aggregated forms of tau.

### Phospho-tau (p-tau)

P-tau species were determined using biotin-HT7 as the capture antibody and acceptor beads conjugated to epitope-specific monoclonal antibodies for detection. Global phosphorylation was assessed by using biotin-HT7 as the capture antibody and a pan anti-pThr antibody conjugated to acceptor beads for detection. As there is no standard available for each p-tau and other post-translationally modified tau species, we cannot determine the absolute linearity for each assay. The dilution linearity was tested using the brain lysates of the 16 week old brain samples (data not shown) and the results were used to guide the amount of protein used in the assays.

### Acetylated tau (ac-tau), ubiquitinated tau (ub-tau) and nitrated tau (nY-tau)

To assess tau acetylation, ubiquitination and nitration during aging, biotin-HT7 was used as the capture antibody in combination with either a pan anti-acetylated lysine monoclonal antibody, an anti-ubiquitin antibody or an anti-nY29 tau antibody conjugated to acceptor beads for detection.

### Western blot analysis

The soluble or insoluble fractions of brain extracts were separated on NuPage gels (Invitrogen) and transferred to a nitrocellulose membrane. The antibody used in each blot was as specified. The blots were developed using electrochemiluminescence (ECL) according to the manufacturer’s instruction (Amersham GE HealthCare).

### Statistical analysis

The GraphPad Prism program was used for statistical analysis. Factorial ANOVAs were used to analyze the brain and CSF tau data for male and female mice at various ages with sex and age as the between subjects factors.

## Additional files

Additional file 1: Figure S1. Age-dependent change of pS262, pS400, AT180, PHF13, AT100 and pS409 tau in the brain insoluble fraction from rTg4510 mice. Brain insoluble fractions from animals 6–32 weeks of age (n = 18-20 per group) were analyzed using AlphaLISA based immunoassays. pS262 tau (A.), pS400 tau (B.), AT180 (pT231) tau (C), PHF13 tau (D), AT100 tau (E) and pS409 tau (F) tau were measured using biotin-HT7 for capture and specific p-tau antibodies for detection. Two hundred ng of protein from the insoluble fractions were used in each assay. The Y-axis is the relative fluorescence readout from Envision (PerkinElmer). Compared to 8-week old mice, the changes of p-tau reached statistical significance at ages 20, 16, and 20 weeks of age for pS262, pS400 and AT180 (pT231), respectively. PHF13 tau, AT100 tau and pS409 tau were all significantly elevated at week 16 and 32 as compared to 8-week old mice (p < 0.01). Additional file 2: Figure S2. Correlation of various tau species in the insoluble fraction of rTg4510 mouse brain. The data in Figure 3 and Additional file 1: Figure S1 were analyzed for correlation with PHF6 tau using Prism 5.0 software. Shown are correlations of PHF6 tau with: A. pS262 tau; B. pS400 tau; C. AT180 tau; D. PHF13 tau; E. AT100 tau; and F. pS409 tau. Additional file 3: Figure S3. ac-Tau (K280) antibody reacts specifically to acetylated tau. HEK293 cells were transfected with vector control (Mock), human tau (Tau), or co-transfected with human tau and P300 catalytic domain (Tau & P300). At 24 hours post transfection, the cells were treated with HDAC inhibitors (10 mM sodium butyrate & 1 μM Trichostatin A, 24 hours) or HAT inhibitor C646 [14] (20 μM, 8 hours) as indicated. Ten microgram of cell lysate was loaded for Western blotting to detect total tau with HT-7 monoclonal antibody (1 μg/ml), acetylated tau with K280 polyclonal antibody (1:1000), and acetylated tubulin with an ac-tubulin monoclonal antibody (Sigma, 1 μg/ml) as a control.

## Abbreviations

AD: Alzheimer’s disease
FTDP-17: Frontotemporal dementia with parkinsonism on chromosome 17
NFT: Neurofibrillary tangles
CSF: Cerebrospinal fluid
p-tau: Phosphorylated tau
