# Supplementary material for: Analysis of tau post-translational modifications in rTg4510 mice, a model of tau pathology
Source: Mol Neurodegener. 2015 Mar 26;10:14. doi: 10.1186/s13024-015-0011-1 (PMC4391670; doi:10.1186/s13024-015-0011-1)
Supplement: Additional file 1: Figure S1. — Age-dependent change of pS262, pS400, AT180, PHF13, AT100 and pS409 tau in the brain insoluble fraction from rTg4510 mice. Brain insoluble fractions from animals 6–32 weeks of age (n = 18-20 per group) were analyzed using AlphaLISA based immunoassays. pS262 tau (A.), pS400 tau (B.), AT180 (pT231) tau (C), PHF13 tau (D), AT100 tau (E) and pS409 tau (F) tau were measured using biotin-HT7 for capture and specific p-tau antibodies for detection. Two hundred ng of protein from the insoluble fractions were used in each assay. The Y-axis is the relative fluorescence readout from Envision (PerkinElmer). Compared to 8-week old mice, the changes of p-tau reached statistical significance at ages 20, 16, and 20 weeks of age for pS262, pS400 and AT180 (pT231), respectively. PHF13 tau, AT100 tau and pS409 tau were all significantly elevated at week 16 and 32 as compared to 8-week old mice (p < 0.01). [file 13024_2015_11_MOESM1_ESM.doc]

**Supplement Figure 1**. Age-dependent change of pS262 tau, pS400, AT180, PHF13, AT100 and pS409 tau in the brain insoluble fraction from rTg4510 mice. Brain insoluble fractions from animals 6-32 weeks of age (n=18-20 per group) were analyzed using AlphaLISA based immunoassays. pS262 tau (A.), pS400 tau (B.), AT180 (pT231) tau (C), PHF13 tau (D), AT100 tau (E) and pS409 tau (F) tau were measured using biotin-HT7 for capture and specific p-tau antibodies for detection. Two hundred ng of protein from the insoluble fractions were used in each assay. The Y-axis is the relative fluorescence readout from Envision (PerkinElmer). Compare to 8-week old mice, the changes of p-tau reached statistical significance at ages 20, 16, and 20 week of age for pS262, pS400 and AT180 (pT231), respectively. PHF13 tau, AT100 tau and pS409 tau were all significantly elevated at week 16 and 32 as compared to 8-week old mice (p<0.01),

Supplement Figure 1
