# Supplementary material for: Analysis of tau post-translational modifications in rTg4510 mice, a model of tau pathology
Source: Mol Neurodegener. 2015 Mar 26;10:14. doi: 10.1186/s13024-015-0011-1 (PMC4391670; doi:10.1186/s13024-015-0011-1)
Supplement: Additional file 2: Figure S2. — Correlation of various tau species in the insoluble fraction of rTg4510 mouse brain. The data in Figure 3 and Additional file 1: Figure S1 were analyzed for correlation with PHF6 tau using Prism 5.0 software. Shown are correlations of PHF6 tau with: A. pS262 tau; B. pS400 tau; C. AT180 tau; D. PHF13 tau; E. AT100 tau; and F. pS409 tau. [file 13024_2015_11_MOESM2_ESM.doc]

**Supplement Figure 2.** Correlation of various tau species in the insoluble fraction of rTg4510 mouse brain. The data in Figure 3 and Supplement Fig. 1 were analyzed for correlation with PHF6 tau using Prism 5.0 software. Shown are correlations of PHF6 tau with: A. pS262 tau; B. pS400 tau; C. AT180 tau; D. .PHF13 tau; E. AT100 tau; and F. pS409 tau.

Supplement Figure 2
