# Supplementary material for: Analysis of tau post-translational modifications in rTg4510 mice, a model of tau pathology
Source: Mol Neurodegener. 2015 Mar 26;10:14. doi: 10.1186/s13024-015-0011-1 (PMC4391670; doi:10.1186/s13024-015-0011-1)
Supplement: Additional file 3: Figure S3. — ac-Tau (K280) antibody reacts specifically to acetylated tau. HEK293 cells were transfected with vector control (Mock), human tau (Tau), or co-transfected with human tau and P300 catalytic domain (Tau & P300). At 24 hours post transfection, the cells were treated with HDAC inhibitors (10 mM sodium butyrate & 1 μM Trichostatin A, 24 hours) or HAT inhibitor C646 [14] (20 μM, 8 hours) as indicated. Ten microgram of cell lysate was loaded for Western blotting to detect total tau with HT-7 monoclonal antibody (1 μg/ml), acetylated tau with K280 polyclonal antibody (1:1000), and acetylated tubulin with an ac-tubulin monoclonal antibody (Sigma, 1 μg/ml) as a control. [file 13024_2015_11_MOESM3_ESM.doc]

**Supplement Figure 3:** ac-Tau (K280) antibody reacts specifically to acetylated tau. HEK293 cells were transfected with vector control (Mock), human tau (Tau), or co-transfected with human tau and P300 catalytic domain (Tau & P300). At 24 hours post transfection, the cells were treated with HDAC inhibitors (10 mM sodium butyrate & 1 μM Trichostatin A, 24 hours) or HAT inhibitor C646 (14) (20 μM, 8 hours) as indicated. Ten microgram of cell lysate was loaded for Western blotting to detect total tau with HT-7 monoclonal antibody (1 μg/ml), acetylated tau with K280 polyclonal antibody (1:1000), and acetylated tubulin with an ac-tubulin monoclonal antibody (Sigma, 1 μg/ml) as a control.

**Supplement Figure 3**

**
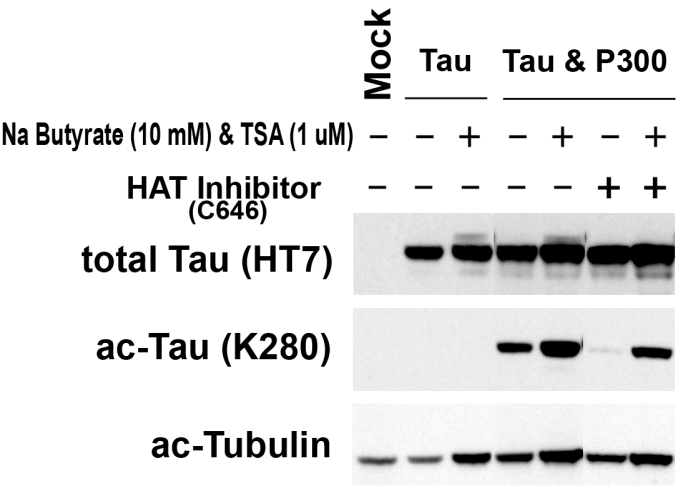
**
